# Defining and measuring population health quality of outpatient diabetes care in Israel: lessons from the quality indicators in community health program

**DOI:** 10.1186/s13584-018-0216-1

**Published:** 2018-05-03

**Authors:** Leonard M. Pogach, David C. Aron

**Affiliations:** Livingston, USA

**Keywords:** Diabetes measures, Population health, Complex conditions, Unintended harms

## Abstract

In Israel, as in other Organization for Economic Co-operation and Development countries, performance measurement is a key public health strategy in monitoring and improving population health outcomes. The Israeli Quality Indicators in Community Healthcare (QICH) program has utilized electronic health records to monitor ambulatory care for the entire Israeli population since 2002. In 2006 the measures were updated to include laboratory values. They have been subsequently revised by stratifying by age, duration, adding medications, and changing frequency of testing for certain process measures. However, the QICH glycemic control measures do not address co-morbid conditions either thru exclusion criteria or higher target ranges. They also do not address potential over treatment in patients with complex medication conditions.

In the United States there have also been changes in nationally endorsed diabetes specific performance measures since 2007. However, there have also been public disagreements among United States professional societies, government agencies, and performance measurement organizations as to whether the current glycemic dichotomous (“all or none”) threshold measures, without exclusion criteria, are consistent with the most recent evidence. Specifically, most guidelines now recommend individualized target goals based upon co-morbid conditions, risk of harms from medications, and patient preferences.

Concerns have been raised that the current glycemic performance measures have resulted in inappropriate care, such as medication over-treatment, and serious harms, such as hypoglycemia, especially in older adults. There currently are no national surveillance systems or measures that monitor these untoward outcomes.

We recommend several actions that QICH could consider to advance diabetes specific performance measurement science and population health: Convene an international conference; implement technical modifications of current measures and surveillance systems; and, most importantly, acknowledge patient autonomy by developing measures that document individualization of target values using shared decision making.

## Background

Since 2002, the Israel Quality Indicators in Community Healthcare (QICH) program has monitored ambulatory care for the Israeli population using electronic health care records. Indicators were created with the consensus of health plans and the Israeli medical association. Diabetes was one of the initial conditions and was expanded in 2006 to include intermediate outcome data [1] similar to the National Committee for Quality Assurance (NCQA) measures approved for implementation in 2007 [2]. The QICH denominator differed by including only those patients receiving oral agents or insulin.

Calderon-Margalit et al. [3] report improved trends in adherence to process outcomes and intermediate outcomes from 2002 to 2010. Since all patients received medications, the improvement is not likely to be attributable to increased prevalence of new-onset diabetes. However, without stratification trends in the < 65 and 65–74-year age groups could not be assessed.

Additionally, there was a positive association of the composite quality indicator score with rates of end-stage complications and mortality from 2006 to 2012, accounting for a lag period. These findings are similar to United States [US] trends [4]. However, unmeasured factors, such as decreased smoking rates or improved care delivery, could be more important than intermediate outcome measures, especially given the short lag time.

QICH continued to utilize the NCQA measures [< 130/80 mm/Hg, < 7% A1c] through 2010 for all patients 18–74 years, although HEDIS revised them in 2008 after early termination of the ACCORD Study. However, after 2012, QICH significantly revised the measures (Ronit Calderon, personal communication). The changes included identification of patients by laboratory value; changing the target values of A1c to < 8% for older patients and those with duration of diabetes greater than 10 years; assessment of patients with nephropathy receiving ACEI/ARB therapy; and changing the frequency of eye examinations from annually to every other year.

## Commentary

What lessons from the QICH experience may be generalized to OCED nations, including the United States (US)?

First, the QICH program provides no financial payments for physicians or plans. Presumably professionalism is the motivation for improved trends, although we cannot exclude peer pressure. This approach warrants careful consideration by US Healthcare leaders.

Second, QICH made evidence-based changes in measures. For example, setting higher A1c targets not only for older patients, but also for those with longer duration diabetes of diabetes, is appropriate with the risk/benefit in these individuals, as first proposed in the 2003 Department of Veterans Affairs/Department of Defense Diabetes (VA/DoD) Guidelines [5]***.*** The use of A1c for inclusion into the measurement cohort can identify undiagnosed diabetes; in the US, about 24% of patients were unaware of or did not report having diabetes in 2015 [6].

However, neither QICH nor US organizations have developed glycemic measures consistent with the most recent guidelines from the American Geriatrics Society [7], the American Diabetes Association [8], VA/DoD [9], and the American College of Physicians [10]. These guidelines all recommend individualized A1c target values of 8.0 to 8.5% (and even 9.0%) for older adults with diabetes based upon the presence of multiple serious complex conditions or poor health/frailty. About half of the US population 65–69 years of age fall into these categories [11]**.** The American College of Physicians has recently recommended that there should be no performance measurement targets in patients for whom avoidance of symptoms may be an appropriate goal [10].

Additionally, the US Department of Health and Human Services published a report that recommended that the < 8% A1c measure for older adults be revisited as part of a national strategy to reduce serious hypoglycemia [12]. The report specifically recommended excluding individuals with co-morbid conditions, stratifying by medications, and including a < 7% A1c balancing measure [13] to identify overtreatment. The NQF endorsed “Optimal Composite Measure” includes < 8% A1c limits exclusion criteria only for death, permanent nursing home, or hospice care.

In contrast, we have proposed [14] that glycemic measures for older patients on hypoglycemic agents with co-morbid conditions should have accountability thresholds only for extremes of control (< 7% A1c and > 9% A1c) and define an acceptable middle range (7.5%–8.5% A1c) for quality improvement consistent with evidence and laboratory science [15].

Finally, neither QICH nor the US have been proactive in developing timely surveillance systems to evaluate harms that can result from poorly specified population health measures-the Black Swan effect [16]. The most concerning example is that marketing of < 7%, which began in 2002, may have contributed to an increase in serious hypoglycemia in the older US population [17]. High rates of < 7%A1c in sicker, older adults on hypoglycemic agents persist in the UA Federal [18] and private [19] sectors.

The challenge for QICH is to translate lessons from Israel and the US into furthering the development of guideline concordant measures that can assess population health while simultaneously respecting the principle of patient autonomy (respect for persons). We propose the following:

1. Israel could consider organizing an international meeting to address diabetes measures, thus taking the lead in reforming diabetes performance measurement.
2. Performance measurement organizations should immediately eliminate the use of “adjectives” to define “measures”. In the US the term “optimal” is used, whereas the QICH measure is deemed “appropriate”-perhaps “recommended” would be more neutral. Adjectives intrude upon the concept of beneficence defined by the individual assessment of benefit and risks.
3. Develop/adapt measures to assess the development of an individualized target A1c goal in lieu of a specific target for older adults including, at least 1) age, 2) comorbidities, and 3) risk for hypoglycemia. This was proposed by the US Centers for Medicare and Medicaid Services in 2016, but not adopted [20]. Substantial evidence supports shared decision making as enhancing patient satisfaction and physician trust are enhanced, even if intermediate outcomes are not improved [9]. The National Quality Forum has recently recommended development of measures for shared decision making [21].

## Conclusion

Balancing benefits and harms of treatment for individuals while monitoring population health will require a major shift in our approach to diabetes performance measurement. It will be necessary, for some measures, to involve patients and clinicians in setting personalized goals rather than being dictated by “meeting a measure”. Treatment decisions require knowledge-guided by professionalism and patient consent, guided by ethics [22]. “The physician should not treat the disease but the patient who is suffering from it (Rambam).”
